# Morphological and anatomical changes during dormancy break of the seeds of *Fritillaria taipaiensis*

**DOI:** 10.1080/15592324.2023.2194748

**Published:** 2023-03-31

**Authors:** Min Luo, Jing Gao, Ran Liu, ShiQi Wang, Guangzhi Wang

**Affiliations:** School of Pharmacy, Chengdu University of Traditional Chinese Medicine, Chengdu, China

**Keywords:** *Fritillaria taipaiensis* P. Y. Li, embryo, seed dormancy, morphology and anatomy

## Abstract

*Fritillaria taipaiensis* P. Y. Li is the most suitable species planted at low altitudes among other species used as Tendrilleaf Fritillary Bulb, whose seeds embracing the morphological and physiological dormancy need to experience a long-dormant time from sowing to germination. In this study, the developmental changes of *F. taipaiensis* seeds during dormancy period were observed by morphological and anatomical observation, and the cause of long-term dormancy of seeds was discussed from the perspective of embryonic development. The process of embryonic organogenesis was revealed during the dormancy stage by the paraffin section. The effects of testa, endosperm and temperature on dormant seeds were discussed. Furthermore, we found that the mainly dormant reason was caused by the morphological dormancy, which accounted for 86% of seed development time. The differentiation time of the globular or pear-shaped embryo into a short-rod embryo was longer, which was one of the chief reasons for the morphological dormancy and played an important role in embryonic formation. Testa and endosperm with mechanical constraint and inhibitors involved in the dormancy of *F. taipaiensis* seeds. The seeds of *F. taipaiensis*, the average ambient temperature of 6–12°C for morphological dormancy and 11–22°C for physiological dormancy, were unsuitable for seed growth. Therefore, we suggested that the dormancy time of *F. taipaiensis* seeds could be shortened by shortening the development time of the proembryo stage and stratification for the different stages of dormancy.

## Research Highlights

- The organ-building process of the rod embryo was revealed during dormancy.
- Morphological after-ripening is one of the main reasons for the seed dormancy of *Fritillaria taipaiensis*.
- The Morphological long dormancy is mainly caused by the long period of proembryo development.

## GRAPHICAL ABSTRACT

**Figure:**
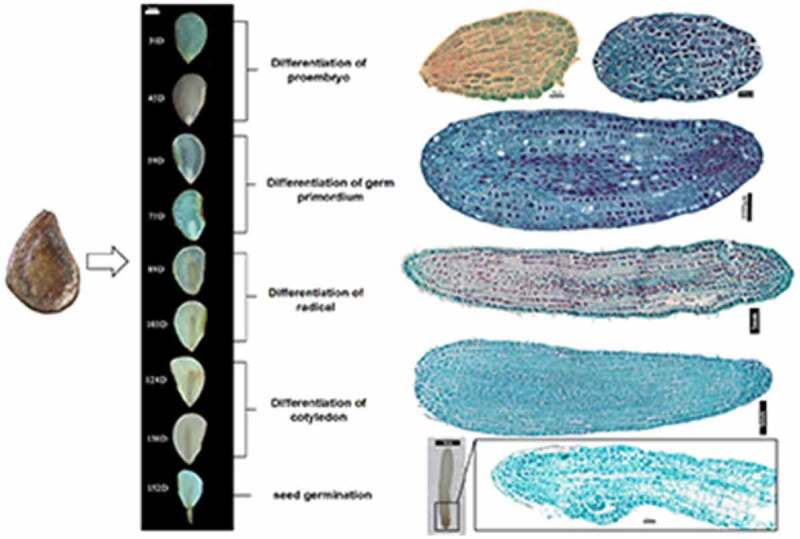

## 1 Introduction

The genus *Fritillaria* L. is one of the largest genera in the Liliaceae family, which comprises approximately about 130 perennial herbaceous species (130–165 taxa) distributed in the temperate regions of the Northern Hemisphere, from Europe, Central Asia to China and Japan^1^. There are 24 species (15 endemics) in China, most of which have medicinal properties^2,3^. *F. taipaiensis*, also known as TaiBei^4^, QinBei^5^, and JianBei^6^, has been used as a herbal medicine with dried bulbs for quite a long time in China. It has good clinical efficacy on cough, bronchitis, unfavorable expectoration^7^ and other diseases. In 2010, it was listed in the Chinese Pharmacopoeia for the first time and was listed as one of the biological sources of Tendrilleaf Fritillary Bulb (Chuanbeimu, Fritillaria Cirrhosae Bulbus)^8^. The wild resources of *F. taipaiensis* are mainly distributed in subalpine areas at 1800 ~ 3150 meters above sea level. They are widely distributed in Sichuan, Chongqing, Hubei, and Shaanxi (Moutain Qinling and its south) in China^9^. Compared with other biological species in Chinese pharmacopoeia, *F. taipaiensis* has the lowest distribution at altitude and is the most suitable for cultivation. The plant can be propagated sexually or vegetatively by seeds or bulbs, respectively. With seed propagation being the main measure adopted in cultivation at low altitudes.

However, the characteristic of seed dormancy gradually is evolved in adaptation to seasons, climates and habitats, which make seeds experience a period of low temperature. The seeds of *F. yuminensis*^10^ and *F. unibracteata*^11^ need to stored for approximately six months before germination. Seed dormancy therefore are one of the major obstacles for the cultivation and domestication. According to Baskin and Baskin^12^ dormancy classification, the seeds of *Fritillaria* belong to morphophysiological dormancy (MPD), which indicates that seeds have both morphological dormancy (MD) and physiological dormancy (PD). That is, small embryos embed in the endosperm of seed and need take a certain amount of time for completing embryo differentiation. When seeds complete morphogenesis, they cannot germinate unless the embryo overcomes the internal inhibitive factor^13^.

Thus, it is necessary to explore the methods of dormancy release for expanding the large-scale propagation of seed agricultural production and meeting the clinical needs for Chuanbeimu. Cultivation is the only way for the sustainable utilization of the resources of *F. taipaiensis* and has achieved satisfying results^14^. This species can be planted at low altitude areas. But there is rarely reported research on the seed cultivation. At present, many studies have focused on understanding the mechanism of seed dormancy such as Arabidopsis thaliana^15,16^, wheat^17^ and other crops in the field of molecular biology. Some achievements have been made on the dormancy mechanism of medicinal plants such as *Paris polyphylla*, *Polygonatum kingianum*^18–20^, *Panax ginseng*^21–23^, and *P. quiquefolium*^24^. In contrast to them, the knowledge about *Fritillaria* seeds still stays at the basic research level. The effective dormancy factors including the temperature, hormone or stratifition play a decisive role for the *Fritillaria* seeds. Y. Gao and Xiao^25^ and Yu, Wei, Chen, Dai, and Yang^26^ set different temperatures to incubate and observe, the result revealed that 15 ~ 20°C and 25°C was the optimum survival temperature for the seeds of *F. delavayi* and *F. cirrhosa* under the stratification. Hu et al.^27^ found 20°C was conducive to germinate for the *F. taipaiensis* seed. And Zhu et al.^10^ indicated that the seeds could finish the MD earlier by imposing exogenous gibberellin (GA_3_), besides proper concentration GA_3_ can replace the cold to reduce time of the PD in some species.

All above showed that the *F. taipaiensis* seeds have good development prospects. However, the studies on growth and germination of *F. taipaiensis* seeds are not enough to determine the key factors of dormancy release and the reasons for promoting germination. During the MD, the *F. taipaiensis* embryo chiefly showed an increase in embryo length, the process of embryo development and organ formation was still unknown. Uncovering the process of morphological development may provide a cue to break the dormancy and germinate. Therefore, the main goal of the present study was to track the morphological and anatomical changes of *F. taipaiensis* seeds by the traditional paraffin section to observe the changes of *F. taipaiensis* seed before germination. We emphasized on embryonic changes and attempted to find a way to break dormancy by associating with the morphological and anatomical changes and dormancy.

## 2 Material and methods

### 2.1 Material

The plant sample was identified as *Fritillaria taipaiensis* P. Y. Li by professor Wang Guangzhi based on the flora of the People’s Republic of China and voucher specimen [No.AZH01433] in the herbarium of the Chengdu Institute of Biology, CAS [CDBI]. The fruit pods of plant samples were provided by Rucaoyuan Chinese Herbal Medicine Planting Company (Sichuan, China). The fruit pods were obtained from plant *F.taipaiensis* and stored in sand at low temperature in June 2021. When fruit pods were decayed in October 2021, we peeled them and got 50 g seeds. These seeds were placed in 3 cm-deph fine mesh polyester bags at the experimental garden for the following experiments, without any manual intervention.

### 2.2 Methods

#### 2.2.1 The appearance and morphology of the seeds

The appearance of 1000 healthy *F. taipaiensis* seeds was directly observed. A total of 30 seeds were randomly selected to record and measured by electronic digital calipers under a stereomicroscope (Stemi 305). Measured Traits include length and width, endosperm length and embryo length of the seeds. Seed length was the longest distance across the seed parallel to the embryo. Seed width of seed was the longest distance across the seed vertical to the embryo. Endosperm length was the distance between the micropylar and the extreme chalazal ends, excluding the peripheral wing of the seed. Embryo length was the radicle to the cotyledonary tip.

Due to seed coat transparency, embryo development could be grasped by transilluminating seeds. Then, 50 seeds which were randomly selected from 2.1 were divided to stationarily observe and measure the percentage of embryo length to endosperm length (E:S ratio), the average germination rate, and the average mildew rate using electronic digital calipers every 7 days under a stereomicroscope (Stemi 305). Three replicate measurements were made. The observation ended until the three measurements showed no significant changes. During the experiment, a wet thermometer was used to record the air temperature every day. A seed was considered germinated when the radicle exceeded the seed coat by 2 mm.

#### 2.2.2 Thousand kernel weight and seed viability assay

Thousand kernel weight (TKW): 1000 fresh seeds were randomly chosen (repeat 3 times) and weighted to calculate the average weight for evaluating the equality and full degree for 1000 seeds.

The seed viability assay^28–30^: The tetrazolium assay (TTC) was used to measure seed viability. Seed viability of *F. taipaiensis* was assessed by slicing longitudinally with a razor and immersion in a 0.5% TTC solution for 24 h at room temperature in the dark. Viable embryos presented a pink or red color. The vigor value of the seeds was calculated according to the color intensity as follows:

Seed viability (%) = number of viable seeds/total number of seeds×100%

  

#### 2.2.3 Embryo development of *F.taipaiensis*

In order to track the embryo development and observe the changes of embryo organs during the dormant period. Ten seeds which came from 2.1 were collected for anatomical study every 7 days. Embryos were obtained by manual dissection under a stereomicroscope (Stemi 305), then soaked in 50%, 70%, 85% and 95% ethanol for 30 min, respectively, and soaked in 100% ethanol and 100% ethanol for 15 min, respectively, to complete dehydration. The dehydrated embryo was cleared in a graded ethanol and xylene series (2:1, 1:1, 1:2) for 30 min/step, then soaked in pure xylene solution twice, 15 min each time^31,32^. After that the transparent embryo is transferred into 1/2 ×ylene+1/2 paraffin, the embryo was soaked in the paraffin in an oven at 45°C overnight, gradually transferred into the pure wax, and the wax was changed twice during the period. Soaking in pure wax for 24 hours, trimming and slicing by using a sliding microtome after drying in oven at 60°C for 25 min. The slices were dewaxed in xylene solution for 1 h, and the xylene solution was changed once during the drying. The slices were soaked in 100%, 100%, 95%, 80%, 70% and 50% ethanol for 2–3 min for rehydration, stained in 1% safranin aqueous solution for 1–2 h, dehydrated in different concentrations of ethanol, and stained with 0.5% fast green for 30–50 s. 95% ethanol and anhydrous ethanol were dehydrated and placed in xylene, and the slides were mounted with neutral gum. The Axiocam 208 color camera was used to observe and photograph^33,34^.

2.2

## 3 Results

### 3.1 Morphology of *F.taipaiensis* seed

**Figure 1.**
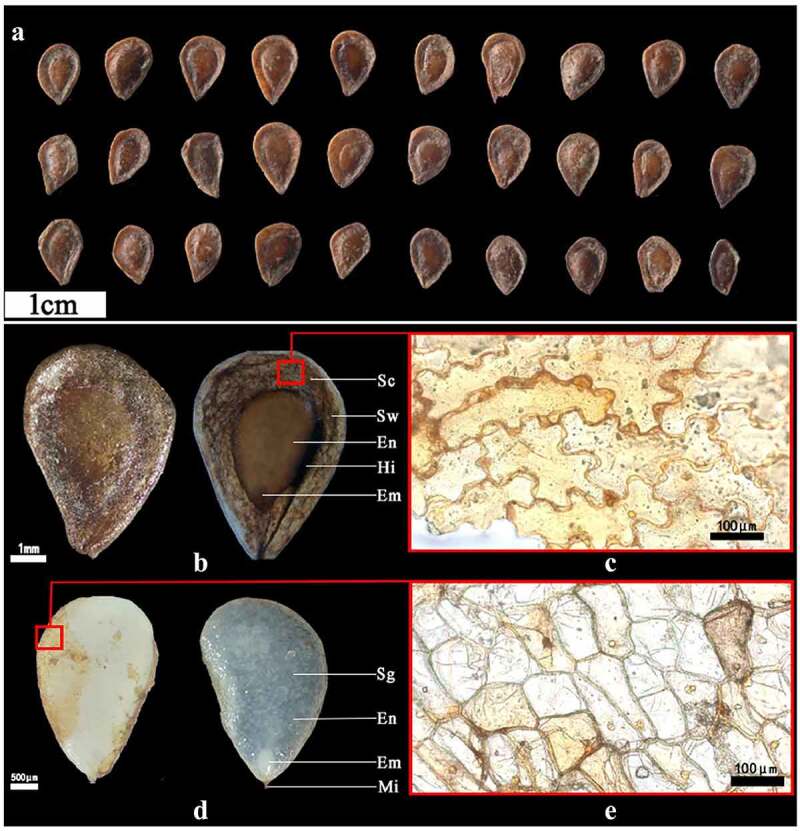
Morphological description of *F. taipaiensis* seeds. The figure c and e were obtained by optical microscope (200× magnification) (a) Seeds in different forms. (b) Seeds in dry (left) and wet (right) states. (c) The testa of the seed under the microscope. (d) Endosperm (without seed coat) in dry (left) and wet (right) state. (e) The endotesta under the microscope [Sw: seed wing. Sc: seed coat. En: endosperm. Em: embryo. Mi: micropyle. Sg: starch granule. Hi: Hilum.].

The seeds of *F. taipaiensis* are brown, flat, smooth, triangular obovate, ovoid or obovate, about 6–7 mm long, 3–4 mm wide (Figure 1a). The vigor value and TKW of the seed were 93% and 3.63 g, respectively. Since the seed coat is transparent, the seed coat, endosperm and embryo can be seen. The endopleura and endosperm adhered strongly. Under a stereomicroscope, the results were observed below after the seed was soaked for 30 s: the seed coat with two layers was seeped into water and was thin and transparent, easy to peel off. The outer testa was brown or brownish yellow, the surface ornamentation was reticular, the epidermal cell was approximately 217.8 μm long and 106.7 μm wide, with irregular shape and disordered and compacted arrangement (Figure 1b–). Endotesta was yellow, closed to endosperm in a dry state, dry and inelastic (Figure 1d). Epidermal cells of endotesta were mostly rectangular or polygonal, closely arranged, without obvious intercellular space, up to 109.1 μm long, about 82.4 μm wide (Figure 1e). The endosperm was milky white about 4.19 mm long and 2.72 mm wide. It was composed of sclerenchyma cells of hemicellulose and contained starch grains arranged closely. The endosperm had no empty cavity and enclosed the embryo tightly. The endosperm layer was thick, so it was difficult to observe the embryo without absorbing water. After absorbing water, the endosperm was translucent with clear cell–cell boundaries, endosperm cells were irregularly shaped and tightly packed (Figure 1d). The embryo was inserted at the base of the endosperm at the end of the seed pore. The embryo was small, 0.68 mm long and 0.37 mm wide, white, dotted or small globular (Figure 1d).

### 3.2 Phenology of embryo growth and germination

**Figure 2.**
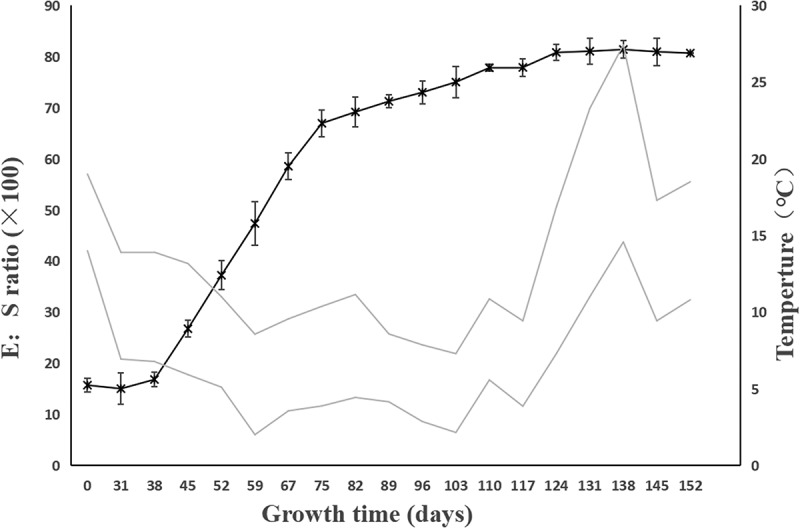
The change of E:S ratio for *F. taipaiensis* seeds. The dotted line indicated the average maximum and minimum temperature of each week. E:S ratio, the ratio of embryo length to endosperm length. Values are means ± SE (*n* = 50).

**Figure 3.**
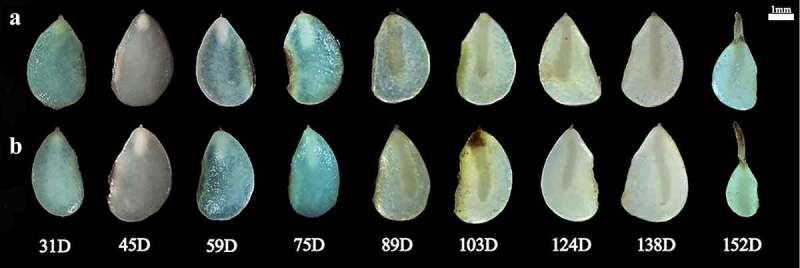
Seed of different development stage (without seed coat). D, the days of seeds growth.

At day 0, the E:S ratio of *F. taipaiensis* seeds was on average 0.162 (Figures 2–3). After incubating 38 days at the average ambient temperature of 9–16°C, the endosperm did not significantly change. Compared with the initial E:S ratio, the E:S ratio at 38 days showed no significant change (*P>0.05*), with the average E:S ratio of 0.173 ± 0.147. The embryos were in the proembryo stage, spherical or pear-shaped, accounting for 25% of the total time of seed growth and development.

When the average temperature of the environment dropped to 5–11°C after 67 days, the seeds were in the stage of rapid development. The starch grains around the embryo were gradually degraded. The endosperm became transparent to provide energy for the growth and development of embryo. The E:S ratio of seeds increased from 0.173 to 0.664. The embryo developed into a short rod by direct observation and in the germ primordium stage by anatomical observation at this time. At 89–124 days, the average ambient temperature was 6–9°C. The endosperm was translucent, more than half of the starch and other substances in the endosperm had been consumed. The endosperm was cellularized, and flocculents were attached around the embryo. The E:S ratio of the seeds increased slowly.

On days 131–152, the average ambient temperature was 11–22°C. The endosperm was transparent, and the seeds began to germinate successively. After day 131, with recording every 7 days and tracking for three times, it was found that the embryo had no significant growth, the E:S ratio exceeded 80% and kept stable, indicating the morphological development was completed (when the E:S ratio accounted for 80% of the endosperm, the seeds were considered to have completed the morphological development). Until 152 days, the average germination rate became steady to show 0.67 ± 0.00 after continuous observation for three weeks, the average moldy rate of seeds was 9.33 ± 1.82 (Table 1)

**Table 1.** The average germination rate and mildew rate of *F. taipaiensis* seeds.

Temperature（℃）		Germination rate	Mildew rate
Maximum	Minimum	（%）	（%）
22	12	0.67 ± 0.00	9.33 ± 1.82

### 3.3 Morphological and anatomical changes during seed development

#### 3.3.1 Embryonic differentiation at the proembryo stage

**Figure 4.**
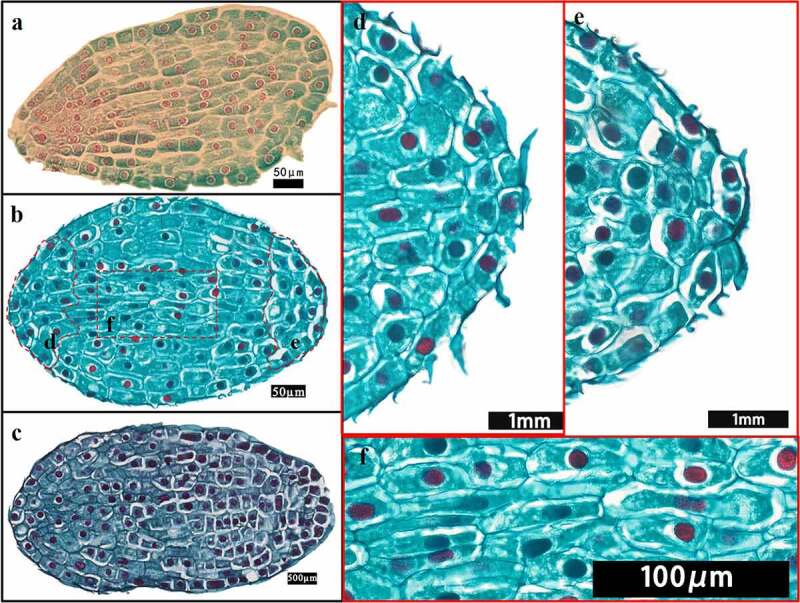
Embryo of the proembryo stage. (a–c) The longitudinal views of embryonic development at the proembryo stage. (d–f) Local enlarged views of the embryo in (b). (a) The embryo of *F. taipaiensis* seed at day 0. (b) The embryo of *F. taipaiensis* was developed for 30 days. (c) Embryos between late proembryo and short rod at 38 days. (d) Cotyledon apex of the proembryo. (e) Radicle region of the proembryo. (f) The center of the proembryo.

The embryo of *F. taipaiensis* at 0 day was composed of 14–16 rows of cells, had more embryonic cells, round nucleus and dense cytoplasm, but no obvious differentiation. It had simple structure, without obvious organ differentiation, spherical or pear-shaped embryo, and was in the proembryo stage (Figure 4a). After 30 days, cells inside embryo gradually divided and differentiated (Figure 4b). Cells near the micropyle gradually became small and dense, and that nucleus occupied almost half of the cell area, which were gradually distinguished from the cells radially developed above (Figure 4be). These cells had stronger meristematic ability and trended to format the region of radicle. During this period, there was no obvious division in the radicle area. The rest of cells inside the embryo mainly divided anticlinally and obliquely, so that the embryo appeared narrow in the radicle area, while the upper part of the radicle was wide and short, like a pear, so it was called “pear-shaped proembryo”. In addition, 5–6 rows of cells in the center of embryo extended radially from the top of the radicle region to the chalazal end, connecting the upper and lower ends of the embryo and playing a role in transportation (Figure 4cf). The outer cells of the embryo gradually lengthened for adapting to the changes in internal cell structure, and formed an epidermogen to protect the embryo^35^. At 38 days, compared with the initial E:S ratio, there was no significant change in E:S ratio (Figure 2), whereas the inner cells of the embryo had been clearly divided into the cotyledon area and the radicle area. In contrast with the rate of cell differentiation in the radicle region, the cells in the cotyledon region divided more rapidly and arranged tightly. The embryo developed into a transitional embryonic morphology between the late proembryonic stage and the short rod embryo, illustrating that the embryo at this time had begun to enter the next developmental stage (Figure 4c).

#### 3.3.2 Differentiation of the germ primordium

**Figure 5.**
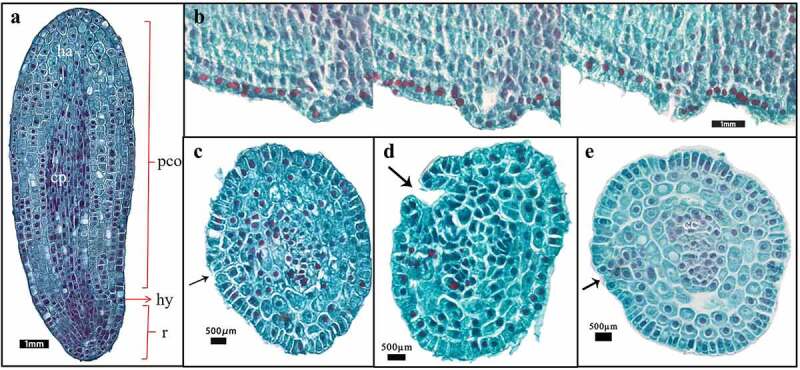
Embryonic development change during the short rod stage. (a–b) Longitudinal sections of embryos. (c–e) Transverse sections of embryos. (b–e) Transverse section between cotyledon base and radicle. (a) Overall view of a short rod-shaped embryo at 67 days. (b) Serial longitudinal section of embryo during groove differentiation. (c) The embryonic edge tissue invaginating and the initial stage of groove appearance. (d) Groove differentiation was completed. (e) Stem meristem cells appeared in the groove. [pco: cotyledon. cp: the cotyledon provascular cells. ha: haustoria. cc: cotyledon coupling. hy: hypophysis. r: radicle.].

The differentiation rate of embryonic cells in different regions was not consistent. The radicle apex cells developed slowly and evolved toward the root cap (Figure 5a). Above the radicle region, the embryo cells divided rapidly in anticlinal and periclinal directions. The number of cell rows added to 21–23, and the embryo body became wider. At days 45–82, the embryo developed into a short rod (Figure 5a). Cells inside the embryo gradually differentiated into the differentiation regions of various organs. Above the radicle apex, the marginal tissue on one side of the embryo was concave inward, a cavity was formed gradually (Figure 5c– arrow) and was closed from top to bottom (Figure 5b) through observation of the transverse and longitudinal section, finally showing a small groove. The groove would form the shoot apical meristem (SAM) in the furture (Figure 5d) and was useful to distinguish the embryonic organ. The development area of hypocotyl and radicle was mainly located below the furrow. Above the furrow was the area of cotyledon differentiation.

As shown in Figure 5a, the cell of cotyledon area proliferated rapidly at this stage. Cells under the epidermal layer of cotyledon apex extended irregularly to absorb nutrition from endosperm. The cells on the side near the primordial germ expanded and specialized into capitate haustorium. The cells in the center of the cotyledon became long narrow and arranged closely, with the flat and long nucleus, which was obviously different from the surrounding cells and specialized to the provascular cells. Provascular cells formed two bundles of vascular, one of which connected the haustorium upward, the other extended downward to the groove, forming the cotyledon coupling (Figure 5d). It was a more mature form of the transport cells at the proembryo stage.

#### 3.3.3 Radicle differentiation

**Figure 6.**
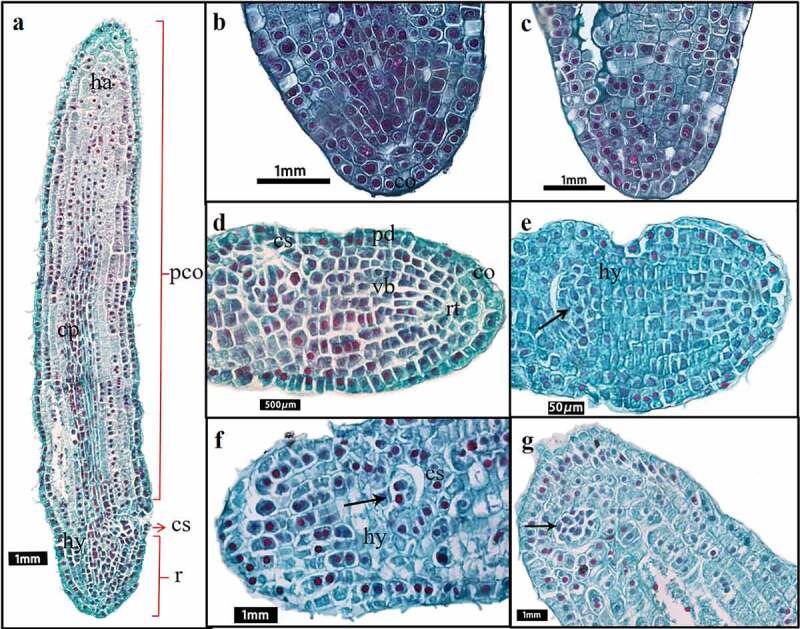
Longitudinal section of the radicle gradually differentiated. (a) A rod-shaped embryo with radicle differentiation completed at 89 days. (b) Radicle of short rod embryo at 67 days. (c) Radicle of the embryo between short rod and long rod at 82 days. (d) Radicle fully differentiated at 89 days. (e) Radicle completed development of embryo morphology at 131 days. (f) The germ primordium emerged gradually and occupied half of the cavity. (g) Germ primordium filled with germ cavity. [pco: the cotyledon. ha: haustoria. cp: cotyledonary provascular cells. hy: hypophysis; cs: cotyledon sheath. r: radicle. pd: protoderm. vb: procambium. co: root cap. rt: the meristematic root. arrow: The shoot apical meristem (SAM)].

At 89–96 days, the embryo was long rod-shaped (Figure 6a), and the radicle elongated slowly. The root tip became sharp from blunt round gradually (Figure 6a–), and the radicle differentiated into root cap, epidermogen, corticogen and stele (Figure 6b). The differentiation of groove was more obvious. With growth of the meristem at the edge of the cotyledon primordium, the furrow was deepened gradually. Finally, a larger cavity, the germ cavity, was formed. Some cells surrounding the germ cavity was a sheath-like extension of the cotyledons, which was the cotyledon sheath and had the function of protecting the germ. The cotyledon sheath was connected with the haustorium by the cotyledon trace, forming the cotyledon coupling.

Meristematic cells with irregular arrangement gradually appeared at the base of embryo cavity as the radicle growth. The meristematic cells, with a large proportion of nuclei, dense protoplasm and strong meristematic ability, were called the germ primordium (Figure 6f–g) which was the precursor of the shoot apical meristem (SAM). At this time, the SAM was not yet fully developed (Figure 6f arrow). As the continuous development of the embryo, the grooves were filled (Figure 6g arrow), which meant the completion of the SAM differentiation. There were 2–3 rows of cells under the germ cavity near the radicle, which were the transitional cells between the coleoptile and the radicle, formed the hypocotyl (Figure 6a). The hypocotyl was beneficial to distinguish the cotyledon, embryo primordium and radicle. It trended to grow downward and promote the elongation of radicle, although the effect was not obvious in *F. taipaiensis* seeds (Figure 6f).

#### 3.3.4 Differentiation of the cotyledons

**Figure 7.**
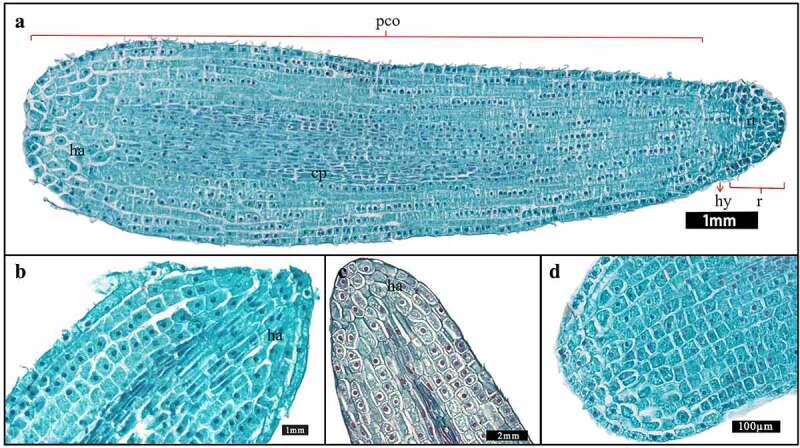
Longitudinal sections of embryos at different developmental stages. (b–d) The partial enlarged views of cotyledons at different stages. (a) Cotyledons developed completely at the linear embryo stage. (b) Cotyledon tips of short rod-shaped embryos. (c) Cotyledon tip of long rod-shape embryo. (d) The cotyledon tip of a fully developed embryo. [pco: the cotyledon. ha: haustoria. cp: cotyledonary provascular cells. hy: hypophysis. r: radicle. rt: the meristematic root].

During the embryo growth of *F. taipaiensis*, the cells inside the cotyledon mainly embarked the division and differentiation in proembryo stage (Figure 4b). At the middle stage of embryo development, the cotyledon showed a sharp apex (near the chalazal end) because of growing upward (Figure 7b). The cells in the cotyledons divided anticlinally and periclinally to extend the length and width of the embryo, which could increase the contact with the endosperm (Figure 7c). At 117–124 days, embryos developed into linear embryos (Figure 7a). The upper part of the embryo became wide and flat, the middle and the lower part of the cotyledon were narrow and thick. The whole growth and development speed of the embryo became gradually level off (Figure 2). Radicle had no obvious trend of division and elongation at this stage (Figure 7a), whereas the cotyledon inside embryo mainly embarked on the periclinal division. The haustorium in cotyledons still existed, but tended to degenerate. The number of cell rows in the upper part of cotyledon increased, so that the apex of cotyledon was relatively flat (Figure 7d).

At 131 days, the embryo basically stopped growing. The embryo was a linear embryo and located in the center of endosperm. The radicle at the micropyle had changed into a conical shape, the germ cavity was almost filled with SAM. The cotyledon base was oblate, the cotyledonary tip was slightly flat. The embryo had obviously differentiated into cotyledon, SAM, radicle, hypocotyl and other organs, which indicated that the embryo of *F. taipaiensis* had developed completely at this time.

#### 3.3.5 Period of seed germination

**Figure 8.**
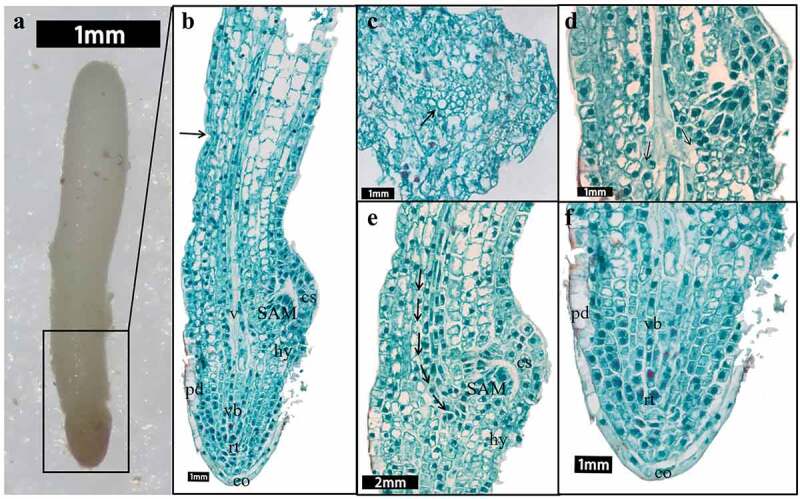
Photographs of embryos and longitudinal sections of embryos at germination stage (expect c). the box indicated a part of the embryo which had broken through the seed coat and exposed outside, the pictures of b-f mainly around the embryo in the box. (a) The embryo of the germination stage. (b) Longitudinal section of the embryo in the box, the arrow: the critical point of the embryos exposed outside the endosperm. (c) Cross-section of radicle, arrow: vascular bundles with thickened cell walls. (d) The vascular bundles became annular vessel. (e) Embryo between cotyledonary base and hypocotyl, arrow: the vascular bundles link to SAM. F: radicle at germination stage. [cs: cotyledon sheath. v: vascular. SAM: the shoot apical meristem. hy: hypophysis. pd: protoderm. vb: procambium. co: root cap. rt: the meristematic root.].

The fully developed seeds completed germination at 152 days according to integrate results of the average germination rate and direct observation. The results of comparison between pre-germinating embryo and post-germinating embryo are as follows: The structure exposed outside the endosperm consisted of a radicle and a convex structure by the stereomicroscope (Figure 8a–b). Contrast with the cotyledons that had completed MD, the cotyledonal morphology after germination had no obvious changes. Only a bundle of central vascular bundles in the middle and lower part of the cotyledon were specialized into the annular vessels (Figure 8b–d), which were early transport organs and existed in the early development stage of plants, with the characteristics of thickened cell wall (Figure 8c), disappearance of protoplasm. The annular vessels diverged slightly at the base of the cotyledon to form two vascular bundles, one slightly curved toward the cotyledon sheath, the other extended toward the hypocotyl to connect with the radicle (Figure 8d). Such differentiation could more accurately deliver nutrients for the growth of SAM and radicle (Figure 8e). Furthermore, the volume of root cap cells increased, the intracellular substances decreased, and the root cap wrapped most of the root apex tissue to protect radicle (Figure 8f). The epidermal cells of the embryo changed more obviously. The cells of the embryonic epidermis layer embedded in the endosperm were long columns. The epidermis exposed outside the endosperm did not change significantly. Epidermal layer adjacent to the root cap became wider and thicker like the root cap cells, which enhanced the function of protecting the radicle. There was no obvious change for the epidermal layer, cortex and mesostele inside radicle.

As shown in Figure 8a, the hypocotyl cells located between SAM and radicle did not proliferate and divide obviously. The cells near the cotyledon sheath located between the base of the cotyledon and the hypocotyl, without obvious differentiation, which were the epicotyl and had strong meristem ability. The cells of this part were prone to divide upward. It was speculated that the power of seed germination for the *F. taipaiensis* was mainly through the division and elongation of epicotyl cells to push the convex part (SAM), unelongated and shorter hypocotyl and radicle to break through the seed coat.

## 4 Discussions

### 4.1 The relationship between seed appearance, seed growth and development

The seed with MPD needed experience a period of physiological dormancy after finishing morphology^12^. Embryo must overcome the seed itself inhibitions to break PD and germinate. The seed-covering layer(s) mainly involved the dead outer testa or pericarp and the living inner testa, endosperm or the radicle^36^. The former acted the mechanical constraint, the latter could involve in the PD. The outer testa of *F. taipaiensis* seed had advantages of being as thin as cicada wings (Figure 1c) and good in water permeability (Figure 1b) under the stereomicroscope. There was an interspace between the micropylar endosperm and outer testa. The above characteristics made the seed different from really hard-coated seeds and protected the embryo from external damage^36^. Besides, the inner layer cells of the seed coat were closely arranged and clung to the endosperm (Figure 1d–e), the embryo had to break the mechanical constraints from endosperm and inner testa before radicle emergence. Therefore, three restrictions form outer testa, inner testa and endosperm must be broken by radicle to germinate

In addition, because the extract of *F. cirrhosae* seed coat^26^ inhibited the germination of Chinese cabbage, it suggested that the seed coat of *F. taipaiensis* contained inhibitors of seed germination.

### 4.2 Relationship between endosperm and seed development

The endosperm played an important role in embryo growth. During the morphogenesis period, endosperm promoted the development of embryo at a range of temperatures. At day 38–67 of seed development, an average ambient temperature of 5–11°C, the E:S ratio of the seeds increased from 0.173 to 0.572. It cued that the embryo growth potential reached peak. The rapid growth processes usually associated with environment signals (such as temperature, light, water, soil, etc.) or internal hormones. Besides, it might also be caused by the following two reasons base on the result of morphology and anatomy:

Reason 1: The cellularization of the endosperm was accelerated. The endosperm was not only the power source for the embryo growth, but also playing a role of prerequisite for embryonic expansion by providing a space. The research of Jacobsen and Pressman^37^ was well proved this point. Even it proposed the endosperm achieved the autolysis by releasing hormone from the embryo in response to light. And the hormone might be a gibberellin. Then the guess had been confirmed at the following studies. Walker et al.^13^ revealed by combined innovative imaging and embryo growth assays that GA biosynthesis was associated with embryo. The biosynthesis genes of GA accumulated in radicle^38^. And GA induced mobilization of oil and cell wall mannan mobilization in the endosperm, the role could not be replaced by other hormones. Therefore, when the embryo was in the proembryonic stage from 0 to 38 days, the embryonic organs (such as radicle, hypocotyl, cotyledon, etc.) were undeveloped, which meant the radicle did not appear in embryo and temporarily had not the ability of releasing the biosynthesis genes (Figure 4c). The endosperm contained abundant starch grains and aleurone grains and was less transparent (Figure 3)^39^. During 45–67 days, radicle was gradually evident, the endosperm was gradually transparent. The endosperm around the embryo was cellularized and spread radially (Figure 4c)^37^, the cavity between the endosperm and the embryo was filled with a highly hydrated liquid with certain viscosity^40,41^. This facilitated embryo contact and absorption. The embryo inside endosperm had a space to grow.

Reason 2: The appearance of transport structure played an important role in embryo growth. Enough nutrition from endosperm was irrigated to ensure the embryo growth. Before day 38, the reason that the rate of division and differentiation of the embryo was relatively slow at proembryo stage might not differentiate conduction structures (Figure 2a–b). Substances exchange between embryo and endosperm depended on the transportation such as suspensor structure, or the epidermal cells with the transport characteristics actively participated in the active absorption of sugar and amino acids in the endosperm^42,43^. However, the suspensor only existed in the early stage of embryonic development, and gradually degenerated through programmed cell death^44^. The latter did not provide sufficient power source for the embryo to support the rapid growth and development. By contrast with proembryo stage, after day 48, a series of changes occurred in the cotyledon primordium (Figure 5). Haustorium differentiated from the top of cotyledon, the vascular bundles were formed in the embryo, giving the embryo the ability to directly absorb and transport nutrients from the endosperm (Figure 2b–c). The appearance of haustorium and transport structure greatly improved the ability of substance exchange between embryo and endosperm.

### 4.3 Relationship between temperature and seed development

The dormant property of the seed was evolved under the unfavorable ecology and phenology for plant propagation or seedling growth. Changes of environmental signals (water, oxygen, temperature, etc.) effected dormant state^12;13^. The seed of *Fritillaria* with MPD could be affected by temperature. The suitable cold/warm stratification was positive for breaking dormancy. Furthermore, the fact that *F. delavayi* seeds had to be stratified until December of the following year before germination indicated that seeds at inappropriate temperature would enter into secondary dormancy and failed to germinate until the appropriate temperature conditions were met again.^25^. This suggests that the seeds first receive environmental signals, which determine seed germination or dormancy. This idea is also supported by the fact that dry seeds need to reach a threshold that initiates embryo growth and germination^45^. So there were scholars carrying out variable-temperature experiments on *Fritillaria* seeds, and finding the following results: e.g. the seeds of *F. przewalskii* within the temperature range of 10°C to 25°C well lived at 15°C^46^. The radicle was too thin to strengthen seedlings after germination. The seeds of *F. unibracteata* could germinate at the range of 5°C to 25°C^47^. Seeds of *F. delavayi* germinated well at 15–20°C and radicle had no emergence at 5°C^25^. The span of temperature expounded that *Fritillaria* seeds of the different species had their own suitable temperature range due to the influence of habitat. So the trail about temperature was necessary to explore for germination of *F. taipaiensis*.

In actual production, seeds of *F. taipaiensis* sowed in late October (late autumn/early winter) and germinated in warm temperatures in April and May under the natural environment. ^12,48^ This was consistent with the rhythm of seed growth in this paper. The E:S ratio and observation (Figures 2–3) showed that seeds of *F. taipaiensis* completed morphological dormancy before the end of spring and germinated in early summer. It was pointed out that *F. taipaiensis* seeds needed low temperature environment to break MD under natural conditions. The property of *F. tubiformis* which radicle protrusion after an incubation for 5 months at 4°C also supported the view^39^. By contrast with temperature during the MD, the higher temperatures could promote germination and PD release. The characteristic of *F. taipaiensis* seed could be released by covering plastic film to shorten the emergence time^49^. It also hinted that the warm stratification was positive to radicle emergence. The studies from Hu et al.^27^ and W. Zhang et al.^50^ also demonstrated the point. The optimal temperature range was 15–20°C for the seed of *F. taipaiensis*, the embryo growth was awfully slow under low temperature (such as 5°C). However, these researches were controlled at a constant temperature. To sum up, we reasonably speculated: Did the seeds of *F. taipaiensis* have the corresponding optimum temperature in different dormancy stages? This might be one of the methods to consider further shortening seed dormancy by stratification. At least for the seeds of *F. taipaiensis*, the average ambient temperature of 6–12°C for MD and 11–22°C for PD were not suitable for seed growth on the basis of the lower germination rate of seeds.

### 4.4 Reasons for longer seed dormancy based on morphology and anatomy

It took 152 days from sowing to the end of seed germination. The time of MD release (131 days) accounted for about 86% of the seed growth and germination time, which was the main reason for the long dormancy period of seeds. Whereas the time for globular or pear-shaped embryos to form short-stem embryos accounted for 29% of the time of MD. During this period, the embryo had not suspensor, the areas of organ differentiation and primordial germ layer. After 30 days (Figure 4a), there were growing up the evident organ differentiation regions or the polarization and axialization of proembryo inside embryo (Figure 4d–f). Based on the hypocotyl as the coordinate cell, the embryonic cells followed the apical-basal pattern associated with auxin polar transport to achieve division and tissue differentiation. Finally, the characteristics about the formation of stem and radicle structure and the separation of apical and basal regions implied the end of proembryo stage (Figure 4c)^51–53^. Therefore, compared with the differentiation time of other organs, finishing the above works might be one of reasons why the longer MD time of *F. taipaiensis* seeds for proembryo (Figures 2–4). We can consider finding a way of shortening the proembryo time to reduce the dormancy of *F. taipaiensis.*

In addition, the inner cells at the apex of cotyledon showed a tendency of lateral expansion. It implied that the proembryo was looking for an effective transport organ to replace the suspensor to absorb endosperm materials, such as the formation of haustorium. The proembryo may exist the possibility of establishing new communication organs to promote the connection between endosperm and embryo, except for establishing key embryonic patterns and developing according to inherent procedures.

## 5 Conclusions

To sum up, this study focused on the anatomical and morphological changes of the embryos of *F. taipaiensis* before germination. We revealed that embryos of *F. taipaiensis* needed to undergo the process of proembryo developing into short rod, long rod and linear embryo, which represented the stage of embryo model establishment, cotyledon sheath and radicle, the SAM and cotyledon structure completion, respectively. We analyzed that the development time of embryo at proembryo stage was one of reasons for the longer dormancy time of seeds, and other reasons might be the endogenous inhibitors in endosperm and seed coat. Furthermore, temperature as one of the environmental factors could effect the embryo growth. Considering the habitat of *F. taipaiensis* seed, the optimal straficational temperature may promote germination. These results provided a clue for further research on promoting seed dormancy breaking, and provided a new idea for seed germination and dormancy breaking of *F. taipaiensis* and even other species ingenus *Fritillaria*.

## Authorship

Min Luo designed and wrote the manuscript. Jing Gao and Ran Liu organized the figures, ShiQi Wang participated in the experiment. Guangzhi Wang reviewed and edited the manuscript.

## Data Availability

All data generated or analyzed during this study are available from the first author upon reasonable request.

## References

[1] Kandemir N, Çelik A, Ullah F. Comparative micro-anatomical features of endemic fritillaria taxa growing in the Mediterranean region (Turkey). Flora. 2022;290:152049. doi:10.1016/j.flora.2022.152049.

[2] Chen Y, Guo S, Guan Y, Li M, An Y, Liu H.The research progress of medicinal plants Fritillaria. Mol Plant Breed. 2019;17(18):6198–12.

[3] Wu X, Duan L, Chen Q, Zhang D. Genetic diversity, population structure, and evolutionary relationships within a taxonomically complex group revealed by AFLP markers: a case study on Fritillaria cirrhosa D. don and closely related species. Glob Ecol Conserv. 2020;24:e01323. doi:10.1016/j.gecco.2020.e01323.

[4] Liu Y, Chen S. A revision of Fritillaria L. (Liliaceae) in the hengduan mountains and adjacent regions, China (1)-A study of Fritillaria cirrhosa D. don and its related species. Acta Phytotax Sin. 1996;34:304–312.

[5] Xiao P, Jiang Y, Li P, Luo Y, Liu Y. The botanical origin and pharmacophylogenetic treatment of Chinese materia medica Beimu. J Syst Evol. 2007;45(4):473–487. doi:10.1360/aps06113.

[6] Shen L, Zhou N, Fu S-Z, Yi D-Y, Jia H, Chen H-Y, Wu Y-M. [Pharmacognostical study on cultivated Fritillaria taipaiensis]. Zhong Yao Cai. 2014;37:45–49. 25090702

[7] Li P, Ji H, Xu G, Xu L. Studies on the antitussive and expectorant effects of Chinese drug beimu. J China Pharm Univ. 1993;24:360–362.

[8] Wang D, Wang S, Chen X, Xu X, Zhu J, Nie L, Long X. Antitussive, expectorant and anti-inflammatory activities of four alkaloids isolated from bulbus of Fritillaria wabuensis. J Ethnopharmacol. 2012;139(1):189–193. doi:10.1016/j.jep.2011.10.036. 22101082

[9] Wang AW, Liu YM, Zhu MM, Ma RX. Isosteroidal alkaloids of Fritillaria taipaiensis and their implication to alzheimer’s disease: isolation, structural elucidation and biological activity. Phytochemistry. 2022;201:113279. doi:10.1016/j.phytochem.2022.113279. 35728673

[10] Zhu M, Huang J, Yang W, Wang H, Saimaiti P, Wang Y. Study on seed morphological dormancy characteristics in fritillaria yuminensis X. Z Duan Chinese Wild Plant Res. 2019;38:1–10.

[11] Xiong H, Ma Z, Guo H, Yang Z, Zhao C, Yang G. Research progress on wild source plant resources distribution and conservation of Fritillariae cirrhosae bulbus. Chin Tradit Herb. 2020;51:2573–2579.

[12] Baskin CC. Seeds: ecology, Biogeography, and evolution of dormancy and germination. Crop Sci. 2014;40(2):564–565. doi:10.2135/cropsci2000.0009br.

[13] Walker M, Pérez M, Steinbrecher T, Gawthrop F, Pavlović I, Novák O, Tarkowská D, Strnad M, Marone F, Nakabayashi K, et al. 2021. Molecular mechanisms and hormonal regulation underpinning morphological dormancy: a case study using apium graveolens (Apiaceae). Plant J. 108(4):1020–1036. doi:10.1111/tpj.15489. 34510583

[14] Petrić M, Subotic A, Trifunović-Momčilov M, Jevremovic S. Morphogenesis in vitro of Fritillaria spp. Floriculture Ornamental Biotechno. 2012;6:78–89.

[15] Xu Y, Wang Y, Du J, Pei S, Guo S, Hao R, Wang D, Zhou G, Li S, O’neill M, et al. 2022. A DE1 BINDING FACTOR 1–GLABRA2 module regulates rhamnogalacturonan I biosynthesis in arabidopsis seed coat mucilage. Plant Cell. 34(4):1396–1414. doi:10.1093/plcell/koac011. 35038740 PMC8972330

[16] Yin X, Bai Y-L, Gong C, Song W, Wu Y, Ye T, Feng Y-Q. 2022. The phytomelatonin receptor PMTR1 regulates seed development and germination by modulating abscisic acid homeostasis in arabidopsis thaliana. J Pineal Res. 72(4). doi:10.1111/jpi.12797. 35319134

[17] Liu M, Wang Z, Wang C, Pan X, Gao W, Yan S, Cao J, Lu J, Chang C, Ma C, et al. 2022. Identification of the wheat (triticum aestivum) IQD gene family and an expression analysis of candidate genes associated with seed dormancy and germination. Int J Mol Sci. 23(8):4093. doi:10.3390/ijms23084093. 35456910 PMC9025732

[18] Qi J, Wei J, Liao D, Ding Z, Yao X, Sun P, Li X. Untargeted metabolomics analysis revealed the major metabolites in the seeds of four polygonatum species. Molecules. 2022;27(4):1445. doi:10.3390/molecules27041445. 35209244 PMC8874640

[19] Wang Y, Bailey DC, Yin S, Dong X, Fournier-Level A. Characterizing rhizome bud dormancy in polygonatum kingianum: development of novel chill models and determination of dormancy release mechanisms by weighted correlation network analysis. Plos One. 2020;15(4):e0231867. doi:10.1371/journal.pone.0231867. 32353065 PMC7192456

[20] Wang Y, Liu X, Su H, Yin S, Han C, Hao D, Dong X. The regulatory mechanism of chilling-induced dormancy transition from endo-dormancy to non-dormancy in polygonatum kingianum coll.Et Hemsl rhizome bud. Plant Mol Biol. 2019;99(3):205–217. doi:10.1007/s11103-018-0812-z. 30627860

[21] Ha Y-I, Lim J-M, Ko SM, Liu JR, Choi D-W. Sequence variability and expression characteristics of the ginseng (Panax ginseng C.A. meyer) DehydrinGene family. J Plant Biol. 2006;49(3):205–211. doi:10.1007/BF03030534.

[22] Kim J, Silva J, Park C, Kim Y, Park N, Sukweenadhi J, Yu J, Shi J, Zhang D, Kim KK, et al. 2022. Overexpression of the panax ginseng CYP703 alters cutin composition of reproductive tissues in arabidopsis. Plants-Basel. 11(3):383. doi:10.3390/plants11030383. 35161364 PMC8839735

[23] Sathiyaraj G, Lee OR, Parvin S, Khorolragchaa A, Kim Y-J, Yang DC. Transcript profiling of antioxidant genes during biotic and abiotic stresses in panax ginseng C. A. meyer. A Meyer Molecular Biol Rep. 2011;38(4):2761–2769. doi:10.1007/s11033-010-0421-7. 21086178

[24] Sun Y, Niu Y, Xu J, Li Y, Luo H, Zhu Y, Liu M, Wu Q, Song J, Sun C, et al. 2013. Discovery of WRKY transcription factors through transcriptome analysis and characterization of a novel methyl jasmonate-inducible PqWRKY1 gene frompanax quinquefolius. Plant Cell Tissue Organ Cult. 114(2):269–277. doi:10.1007/s11240-013-0323-1.

[25] Gao Y, Xiao Y. Seed germination of Fritillaria delavayi. J Fujian Forestry Sci Technol. 2017;44(2). doi:10.13428/j.cnki.fjlk.2017.02.015.

[26] Yu J, Wei J, Chen S, Dai Y, Yang C. Dormancy and germination characteristics of Fritillaria cirrhosa seed. Chin Tradit Herb. 2008;39:1081–1084.

[27] Hu P, Xia Y, Yang Y, Wu P, Fang Q, Zhou X. Effect of temperature on germination of Fritillaria taipaiensis seeds. Res Practice Chinese Med. 2018;32:7–9.

[28] Renzi JP, Chantre GR, Smýkal P, Presotto AD, Zubiaga L, Garayalde AF, Cantamutto MA. Diversity of naturalized hairy vetch (vicia villosa roth) populations in central Argentina as a source of potential adaptive traits for breeding. Front Plant Sci. 2020;11:189. doi:10.3389/fpls.2020.00189. 32180785 PMC7059640

[29] Rossetto M, Gross CL, Jones R, Hunter J. The impact of clonality on an endangered tree (Elaeocarpus williamsianus) in a fragmented rainforest. Biol Conserv. 2004;117(1):33–39. doi:10.1016/S0006-3207(03)00260-X.

[30] Seo YS, Kim EY, Kim WT. The Arabidopsis sn-1-specific mitochondrial acylhydrolase AtDLAH is positively correlated with seed viability. J Exp Bot. 2011;62(15):5683–5698. doi:10.1093/jxb/err250. 21856645 PMC3223057

[31] Rhie YH, Lee SY, Kim KS, Bekker R. Seed dormancy and germination in Jeffersonia dubia (Berberidaceae) as affected by temperature and gibberellic acid. Plant Biol. 2015;17(2):327–334. doi:10.1111/plb.12235. 25319374

[32] Zhang R, Li C, Fu K, Li C, Li C. An improved method for studying whole sections of late developing wheat grain. Biotech Histochem. 2018;93(7):471–477. doi:10.1080/10520295.2017.1386802. 30403883

[33] Fei R, Sun X, Yang P, Chen Z, Ma Y. Anatomical observation of paeonia lactiflora seeds during stratification process. J Anhui Agric Univ. 2017;48(3):354–359.

[34] Gao J, Liu R, Luo M, Wang G. 2022. The clonal growth in aconitum carmichaelii debx. Plant Signal Behav. 17(1). doi:10.1080/15592324.2022.2083818. PMC9225526 35713121

[35] Breuninger H, Rikirsch E, Hermann M, Ueda M, Laux T. Differential expression of WOX genes mediates apical-basal axis formation in the arabidopsis embryo. Dev Cell. 2008;14(6):867–876. doi:10.1016/j.devcel.2008.03.008. 18539115

[36] Finch-Savage WE, Leubner-Metzger G. Seed dormancy and the control of germination. New Phytol. 2006;171(3):501–523. doi:10.1111/j.1469-8137.2006.01787.x. 16866955

[37] Jacobsen JV, Pressman E. A structural study of germination in celery (Apium graveolens L.) seed with emphasis on endosperm breakdown. Planta. 1979;144(3):241–248. doi:10.1007/BF00388765. 24407254

[38] Topham AT, Taylor RE, Yan DW, Nambara E, Johnston IG, Bassel GW. Temperature variability is integrated by a spatially embedded decision-making center to break dormancy in arabidopsis seeds. Proc Natl Acad Sci U S A. 2017;114(25):6629–6634. doi:10.1073/pnas.1704745114. 28584126 PMC5488954

[39] Carasso V, Fusconi A, Hay FR, Dho S, Gallino B, Mucciarelli M. A threatened alpine species, Fritillaria tubiformis subsp. moggridgei: seed morphology and temperature regulation of embryo growth. Plant Biosyst. 2012;146(1):74–83. doi:10.1080/11263504.2011.557094.

[40] Chateigner-Boutin A-L, Alvarado C, Devaux M-F, Durand S, Foucat L, Geairon A, Grélard F, Jamme F, Rogniaux H, Saulnier L, et al. The endosperm cavity of wheat grains contains a highly hydrated gel of arabinoxylan. Plant Sci. 2021;306:110845. doi:10.1016/j.plantsci.2021.110845. 33775355

[41] Povilus RA, Gehring M. Maternal-filial transfer structures in endosperm: a nexus of nutritional dynamics and seed development. Curr Opin Plant Biol. 2022;65:102121. doi:10.1016/j.pbi.2021.102121. 34801784

[42] Ingram G. Family life at close quarters: communication and constraint in angiosperm seed development. Protoplasma. 2010;247(3–4):195–214. doi:10.1007/s00709-010-0184-y. 20661606

[43] Lafon-Placette C, Kohler C. Embryo and endosperm, partners in seed development. Curr Opin Plant Biol. 2014;17:64–69. doi:10.1016/j.pbi.2013.11.008. 24507496

[44] Zhao P, Zhou X-M, Zhang L-Y, Wang W, Ma L-G, Yang L-B, Peng X-B, Bozhkov PV, Sun M-X. A bipartite molecular module controls cell death activation in the basal cell lineage of plant embryos. PLoS Biol. 2013;11(9):11(9. doi:10.1371/journal.pbio.1001655. PMC3769231 24058297

[45] Bradford KJ. Applications of hydrothermal time to quantifying and modeling seed germination and dormancy. Weed Sci. 2002;50(2):248–260. doi:10.1614/0043-1745(2002)050[0248:Aohttq]2.0.Co;2.

[46] Chang Y, Chen Y, Guo F, Lin Y, Tian L. A study on soaking and germination characteristics of Fritillaria przewalskii seeds. Acta Hortic Sin. 2010;19:41–46.

[47] Ma Y, Jin L, Luo G, Han H, Lei D. Preliminary studies on breaking dormancy and stimulating germination of F.Unibracteata Hsiao.et.K.C. Hsia. seeds. J Tradit Chin Med. 2012;27:3214–3217.

[48] Copete E, Herranz JM, Ferrandis P, Baskin CC, Baskin JM. Physiology, morphology and phenology of seed dormancy break and germination in the endemic iberian species narcissus hispanicus (Amaryllidaceae). Ann Bot. 2011;107(6):1003–1016. doi:10.1093/aob/mcr030. 21335326 PMC3080619

[49] Luo M. Research progress in medicinal plant fritillaria taipaiensis P. Y. Li. Chinese Wild Plant Res. 2021;40(2):42–56. doi:10.3969/j.issn.1006-9690.2021.02.008.

[50] Zhang W. Study on seed characteristics of Fritillaria Ussuriensis. Asian Pac J Trop Med. 2021;17(6):49–52.

[51] Jenik PD, Gillmor CS, Lukowitz W. Embryonic patterning in Arabidopsis thaliana. Annu Rev Cell Dev Biol. 2007;23(1):207–236. doi:10.1146/annurev.cellbio.22.011105.102609. 17539754

[52] Mironova V, Teale W, Shahriari M, Dawson J, Palme K. The systems biology of auxin in developing embryos. Trends Plant Sci. 2017;22(3):225–235. doi:10.1016/j.tplants.2016.11.010. 28131745

[53] Wabnik K, Robert HS, Smith RS, Friml J. Modeling framework for the establishment of the apical-basal embryonic axis in plants. Curr Biol. 2013;23(24):2513–2518. doi:10.1016/j.cub.2013.10.038. 24291090

